# Lithium monitoring in clinical practice

**DOI:** 10.1192/j.eurpsy.2021.1291

**Published:** 2021-08-13

**Authors:** E. Sciberras, A. Bellizzi, L. Rapa, C. Vassallo, A. Grech

**Affiliations:** Psychiatry, Mount Carmel Hospital, Attard, Malta

**Keywords:** lithium, monitoring, Psychopharmacology, bipolar disorder

## Abstract

**Introduction:**

Lithium is widely used for the treatment of bipolar disorder. Owing to its narrow therapeutic index and side-effect profile, regular monitoring is recommended by all major guidelines on lithium use.

**Objectives:**

The aim of this study was to determine whether routine lithium monitoring practice at the local mental hospital in Malta reaches the standard set by the most recent NICE guidelines (NICE, 2014a).

**Methods:**

All patients on lithium maintenance treatment for bipolar disorder at the local Mental Hospital were included. Blood tests within the last one year were collected using iSOFT clinical manager (iCM). After the first audit cycle, a lithium monitoring sheet was created in accordance with the NICE guideline and after 6 months of implementation, the second audit cycle was conducuted.

**Results:**

In the first cycle, 28 patients met the NICE criteria for increased risk of toxicity and have a recommended testing frequency for lithium levels of every 3 months. However, only 1 patient was observed to meet this criteria. When assessing the last lithium level only 35.7% were within 0.4-0.8 mmol/L. In the second audit cycle, 28 patients met the NICE criteria for increased risk of toxicity and have a recommended testing frequency for lithium levels of every 3 months. Almost half of the patients (12 patients, 42%) were to observed to meet this criteria. When assessing the last lithium level, 50% were within 0.4-0.8 mmol/L

**Conclusions:**

After the introduction of the lithium monitoring sheet, monitoring improved substantially especially in high risk patients. Moreover, the majority of test results for lithium levels were within the therapeutic range.

